# Syncytial nets vs. chemical signaling: emerging properties of alternative integrative systems

**DOI:** 10.3389/fcell.2023.1320209

**Published:** 2023-12-06

**Authors:** Leonid L. Moroz

**Affiliations:** ^1^ Department of Neuroscience, University of Florida, Gainesville, FL, United States; ^2^ Whitney Laboratory for Marine Bioscience, University of Florida, St. Augustine, FL, United States

**Keywords:** ctenophora, porifera, placozoa, cnidaria, nervous system evolution, neurotransmission, synapses, animal origins

## Introduction

**FIGURE 1:**
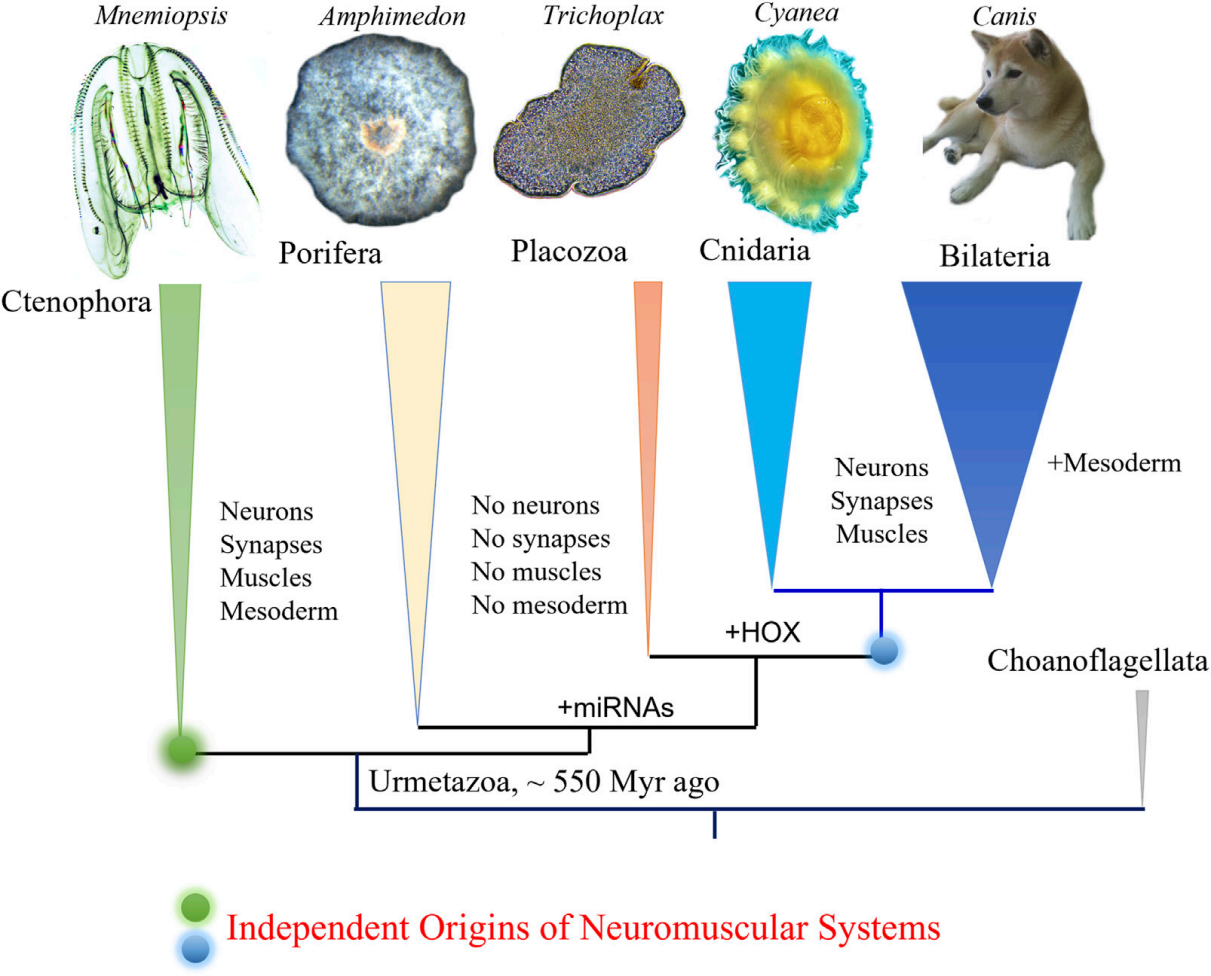
Phylogeny of animals with ctenophores as the sister lineage to the rest of Metazoa. Comparative analyses suggest convergent evolution of neurons, synapses, muscles, and mesoderm (see text for details). Possible origins of microRNA and HOX gene cluster are indicated (Moroz et al., 2014).

Ctenophores or comb jellies possess one of the most unique neural organizations of enigmatic origins; and there are no recognized homologies to any other phylum. The recent integrative (Moroz et al., 2014) and comparative genomics (Ryan et al., 2013; Whelan et al., 2015; Whelan et al., 2017; Li et al., 2021; Whelan and Halanych, 2023), especially cross-phyla chromosome level synteny (Schultz et al., 2023), analyses strongly confirmed a surprising hypothesis that morphologically and behaviorally complex ctenophores are descendants of the earliest metazoan branch, followed by simpler nerveless sponges (Porifera) and Placozoa (Figure 1). Moreover, the molecular deciphering of neural toolkits in ctenophores reveals their unique molecular organization (Moroz, 2015), including reduced representation of canonical bilaterian neurogenic and synaptic gene complement, distinct molecular profiling of ctenophore neurons as well as the apparent lack of classical low molecular weight transmitters (Moroz et al., 2014; Moroz et al., 2020). It is possible to state that ctenophores use remarkably different chemical language for intercellular communications with a unique (mostly unknown) subset of signal molecules as the hallmark of their neural architecture.

Specifically, both the complement of neurotransmitter synthetic enzymes and, most importantly, direct microchemical analyses of neurotransmitters themselves (Moroz et al., 2014; Moroz et al., 2020) indicate that acetylcholine, serotonin, dopamine, noradrenaline, adrenaline, and histamine are not produced by ctenophores studied so far, including *Pleurobrachia* and *Mnemiopsis* (Moroz et al., 2014; Moroz and Kohn, 2016). Furthermore, initial pharmacological tests also failed to observe noticeable behavioral effects of these low molecular weight “classical” transmitters (Moroz et al., 2014; Norekian and Moroz, 2023). Thus, we concluded that monoamines and acetylcholine are true bilaterian innovations (Moroz and Kohn, 2015; 2016; Moroz et al., 2021b), later confirmed with the additional comparative survey of synthetic and metabolic enzymes (Goulty et al., 2023). Glutamate was initially proposed as a neuromuscular transmitter and a possible interneuronal transmitter in ctenophores (Moroz et al., 2014; Moroz, 2015; Moroz et al., 2020; Moroz et al., 2021a). In contrast, ctenophores (including *Pleurobrachia* and *Mnemiopsis* with two sequenced genomes at that time) developed several dozen small signaling peptides and neuropeptides, which have no detectable homologs outside Ctenophora (Moroz et al., 2014; Moroz and Kohn, 2016) with two possible exceptions (Yañez-Guerra et al., 2022).

The obtained interdisciplinary evidence leads to the conclusion that ctenophores independently developed neural systems (Moroz, 2014; Moroz et al., 2014) and independently evolved synaptic organization (Moroz and Kohn, 2016; Moroz et al., 2021b). Therefore, ctenophore neurons are not homologous to cnidarian and bilaterian neurons. Thus, we attempted to refine and broaden the definition of neurons and also used terms of alternative neural and integrative systems (Moroz et al., 2021b; Moroz and Romanova, 2022). In other words, *neurons are synaptically coupled polarized and highly heterogenous secretory cells at the top of behavioral hierarchies with learning capabilities*; and we postulated that neurons are functional rather than genetic categories (Moroz and Kohn, 2016).

**FIGURE 2:**
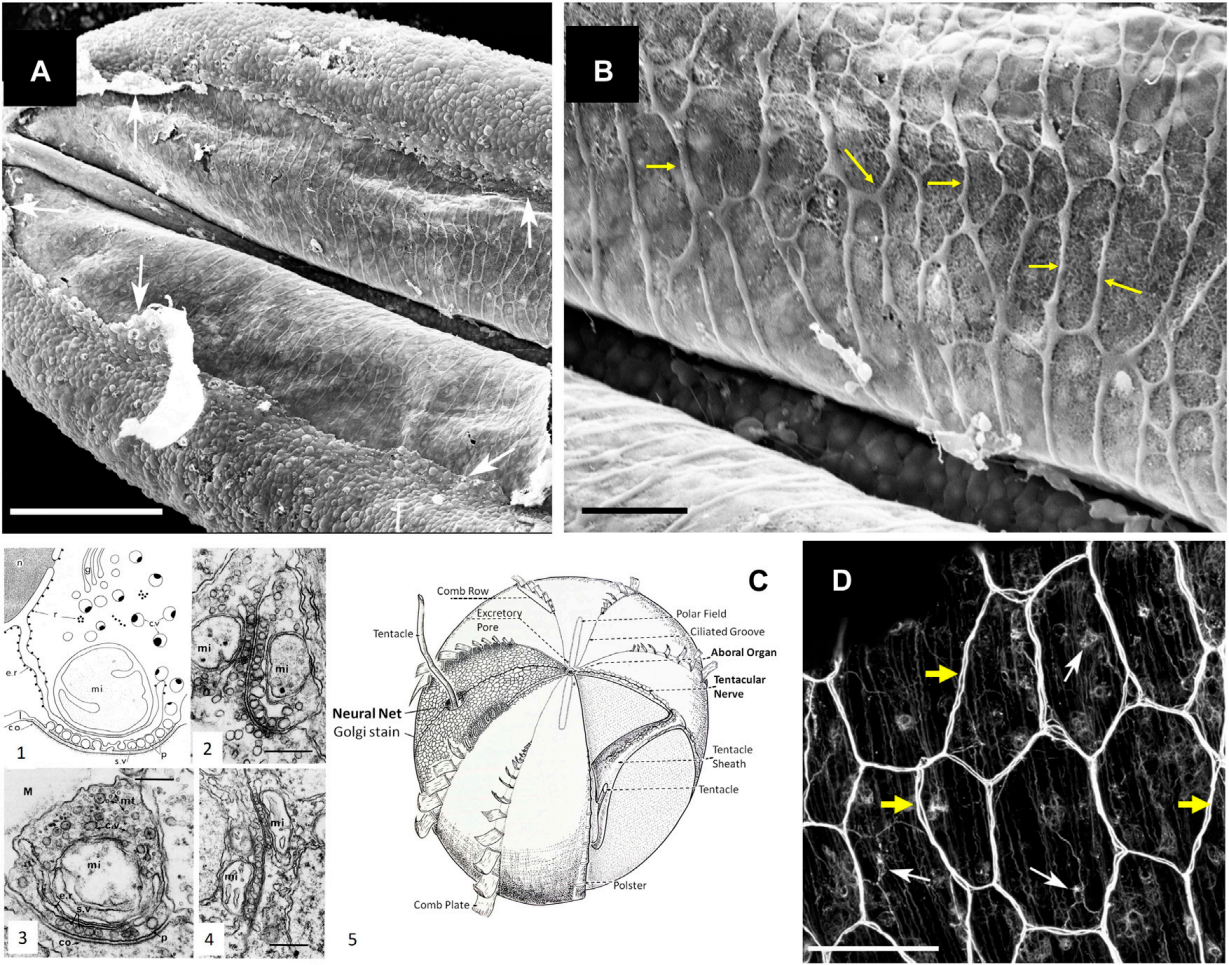
Syncytium-like neuroid systems in tentacles of *Pleurobrachia bachei* (from (Norekian and Moroz, 2019b)). Scanning electron microscopy of the polygonal neural net in the tentacle pocket. **(A)** The upper epithelium layer is peeled off in some areas (arrows), revealing the underlying layer of the neural network. **(B)** The neural syncytial-type network consists of different polygonal units, neural cell bodies, and anastomosed neural processes (yellow arrows). **(C)** Neural nets and unique synaptic organization in ctenophores (modified from (Hernandez-Nicaise, 1991; Moroz, 2015)). (1) The basic features of synapses in ctenophores. The generalized asymmetrical synapse. (2) Symmetrical neurite-to-neurite synapse in *Beroe*. Scale bar: 100 nm. (3) The asymmetrical synapse between a neurite and an epithelial cell (ep) in the epidermis of *Pleurobrachia*. Scale bar: 200 nm. (4) Soma-to-soma reciprocal synapse in the epithelium of *Bolina hydatina*. Scale bar: 100 nm. c.v., cytoplasmic vesicles; co, dense coat on the postsynaptic membrane; e.r., endoplasmic reticulum; g, Golgi complex; l, intracleft dense line; M, mesoglea; mi, mitochondrion; mt, microtubules; n, nucleus; p, presynaptic dense projections; r, ribosomes; s.v., synaptic vesicle. (5) The schematic diagram of the subepithelial nerve system of a generalized cydippid (the aboral view). Images are reproduced and adapted from Hernandez-Nicaise (Hernandez-Nicaise, 1991) with permission from Wiley-Liss, Inc. **(D)** Subepithelial neural net in *Pleuronrachia bachei* stained with tubulin antibody (from (Norekian and Moroz, 2019b)). Neural net consists of polygonal units of different shapes and sizes. There are individual neurons (white arrows) with clearly visible neurites (yellow arrows). The segments of the neural net are composed of tightly packed thin processes, rather than a single thick axon.Scale bars: **(A)** 100 μm; **(C)** 20 μm, **(D)** 50 μm.

In summary, ctenophore neurons result from convergent evolution with their very own array of chemical transmitters, including ctenophore-specific neuropeptides. Recent immunohistochemical and pharmacological experiments confirmed this hypothesis and showed specific distribution and behavioral effects of ctenophore-specific neurotransmitters in *Mnemiopsis* (Sachkova et al., 2021) and *Bolinopsis* (Hayakawa et al., 2022). The overall assessment was that ctenophores broadly used chemical (volume) and more localized synaptic signaling as the dominant way of interneuronal communications with more than 100 signaling molecules (Moroz et al., 2021b). Earlier transmission electron microscopy data identified unique chemical synapses across structures and species in ctenophores, as summarized by Mari Luz Hernandez-Nicaise (Hernandez-Nicaise, 1991), see also Figure 2C.

## Structural uniqueness of ctenophore neural systems

Recent and remarkable ultrastructural data with volume microscopy validate the uniqueness of neural systems and synapses in ctenophores (Sachkova et al., 2021; Burkhardt et al., 2023), further reinforcing our earlier hypothesis of their independent origins (Moroz and Kohn, 2016). However, besides the canonical neural organization with distinct synapses, ctenophores likely possess syncytial-type connectivity in some neuronal populations, such as components of subepithelial nerve net and possibly in the gut (Burkhardt et al., 2023). This 3D electron microscopy reconstruction of neural nets highlighted an apparent “resurrection” of the original Golgi’s reticular theory (Burkhardt et al., 2023). Furthermore, the initial perception of the novel volume microscopic data might be that non-[chemical]synaptic transmission is the distinct characteristic of ctenophore organization in general (Dunn, 2023; Lenharo, 2023), in contrast to other animals and the Cajal’s neuronal doctrine. Moreover, recent discussions and news releases might represent these ultrastructural data as evidence that all ctenophore neurons form the neuroid-type syncytium and have reduced chemical transmission across all neural circuits. Or this situation might be viewed as the predominance of syncytial organization for electrical propagation of signals vs. chemical transmitter-mediated signaling. Experimental functional exploration is needed to understand the cellular bases of ctenophore behaviors.

Toward this discussion, I think that ctenophore neural communications are primarily chemical, with deep ancestry of chemical signaling at the base of animal and neural organization. Here, I summarize this viewpoint and the prospects for future studies.

## Chemical synapses and signaling in ctenophores vs. direct reticular coupling

The Neuron Doctrine postulated anatomical and functional identities of individual neurons as the foundation of any neural organization, stressing morphological and physiological discontinuity of neurons in central and peripheral neural systems. Nevertheless, in his vision of the Neuron Doctrine, Raymon y Cajal wisely considered that “neuronal discontinuity… could sustain *some exceptions*” (Cajal, 1995; Bullock et al., 2005). Coupling cells and neurites into functional syncytia might occur with and without electrical synapses. Ctenophores present an exceptional opportunity to readdress 130-year-old concepts of neuronal architectures.

There are three groups of questions. i) How universal are ctenophore neural syncytia during development and across species? ii) Is syncytial organization unique to ctenophore neurons? iii) What are relationships between neuroid syncytia and chemical signaling with distinct secretory machinery in behavioral integrations of ctenophores? Interdisciplinary comparative studies would be needed to address these questions experimentally.

Burkhard and others performed their remarkable 3D electron microscopy observations on small, just-hatching larval/juvenile animals of the lobate ctenophore, *Mnemiopsis leidyi* (Sachkova et al., 2021; Burkhardt et al., 2023), with developing neural systems consisting of a few dozen putative neurons (Norekian and Moroz, 2021). Whether or not the syncytial organization is preserved within a greater neuronal diversity in adult *Mnemiopsis* must be determined.

First, the neural syncytium within some ctenophore neural nets is possible and likely exists in other species, such as the cydippid *Pleurobrachia bachei* (e.g., Figure 1C in (Moroz et al., 2014)). For example, we did observe such architecture within the nerve net of tentacle pockets (Norekian and Moroz, 2019b) of adult *Pleurobrachia* (Figures 2A,B), the species with an estimated ∼10,000 individual neurons (Norekian and Moroz, 2016; 2019b). Nevertheless, most subepithelial neural nets in *Pleurobrachia* and more than ten other investigated species have neurons with two or more neurites within their orthogons (Figure 2D, see details in (Norekian and Moroz, 2016;2019b;a;^2020^), and in contrast to one neurite of studied microscopic *Mnemiopsis*, suggesting that different types of communications are involved.

Second, although syncytial types of networks are relatively rare, neuroid-type syncytia, similar to these found in ctenophores, were observed in the representatives of at least six animal phyla. However, this list can be expanded since most “minor” phyla remain unexplored. Syncytial-like neural nets might exist in the colonial polyp *Velella* (Mackie, 1960; Mackie et al., 1988) [Cnidaria]. In the cephalopod stellate ganglion, neuronal processes are fused to form giant axons (Young, 1939). Neuronal membrane fusion was also reported in gastropod molluscs, annelids [leeches], nematodes, and mammals (Oren-Suissa et al., 2010; Giordano-Santini et al., 2016; Giordano-Santini et al., 2020). Specifically, neurite and synaptic fusion occur during neural development and neuroplasticity in *Drosophila* (Yu and Schuldiner, 2014) [Arthropoda] and mammals (Faust et al., 2021) [Chordata], likely contributing to metabolic coupling, fast propagating, axon and dendrite pruning, and integration of signaling.

Third, based on published data, only a limited fraction of ctenophore neurons make a syncytial nerve net (Burkhardt et al., 2023). In the recent reconstruction, only 5 of 33 studied neurons in the early stages of *Mnemiopsis* can form a syncytium with fused plasma membranes (Burkhardt et al., 2023). Still, these characterized neurons revealed diverse chemical synapses with characteristic ctenophore-specific presynaptic triads of organelles arranged in layers of synaptic vesicles, endoplasmic reticulum, and mitochondrion (Hernandez-Nicaise, 1973; Hernandez-Nicaise, 1991).

Burkhard and others did not report chemical synapses between subepithelial neurons; however, 3D reconstruction revealed chemical synapses from subepithelial neurons to multiple effector cells such as ciliated structures—polster cells in combs (Burkhardt et al., 2023). Furthermore, 4 of 5 studied populations of sensory neurons make morphologically recognized synapses to subepithelial and mesogleal neurons as well as among themselves and comb cells (Burkhardt et al., 2023), confirming the widespread distribution of chemical synapses within neural systems of *Mnemiopsis*.

It is worth noting that all ctenophore neurons and their neurites contained a diversity of secretory vesicles, suggesting recruitments of multiple neurotransmitters with possible co-localization of signal molecules within the same neuron. The presented ultramicroscopic images indicate about 60–70 sites with dense-core vesicles within a 2-3 neuronal soma diameter area (Burkhardt et al., 2023), suggesting that even this anastomosed subnet can be a neurosecretory system without identified gap junctions among subepithelial neurons. Indeed, the anastomosed neurites contain endogenous neuropeptides, e.g., ML02736a (Sachkova et al., 2021) as possible secretory products of these nets.

Structural constraints of the discovered syncytial-like net are equally essential in understanding the directional propagation of neural signaling in ctenophores. Burkhard and others visualized distinct “blebbed or ‘pearls-on-a-string’ morphology” of neurites in the subepithelial layer with a chain of secretory vesicles (Burkhardt et al., 2023). Of note, secretory vesicles are separated by extremely narrow (∼50–60 nm) cytoplasmatic bridges, sufficient for few microtubules to pass through. A similar type of organization was also recently observed in some rodent axons (Griswold et al., 2023). How these vesicles are transported to these locations or maintained is unclear. How, for example, electrical signals can be propagated along these ultranarrow channels with apparently high resistance are unanswered questions. Saltatory electrical conduction combined with the volume release of neurosecretory molecules might occur. Unfortunately, the majority of signal molecules are unknown in the ctenophore lineage. The current subset of transmitters includes i) L-/D Glutamate (Moroz et al., 2014; Moroz et al., 2020) and ii) glycine as a potential agonist of some ionotropic glutamate receptors in ctenophores (Alberstein et al., 2015; Yu et al., 2016), iii) gaseous nitric oxide (NO) (Moroz et al., 2023), plus iv) several ctenophore-specific neuropeptides (Moroz et al., 2014; Sachkova et al., 2021; Hayakawa et al., 2022), and possibly some catecholamines (Townsend and Sweeney, 2019). Many surprises are expected with apparently alternative chemical “syntax” and even the chemical “alphabet” of signaling molecules in this still very enigmatic lineage of basal metazoans.

## Conclusion

1. Ctenophore nets are structurally and molecularly unique compared to other metazoans. The syncytial-type organization occurs in neural nets within the subepithelium, the gut of *Mnemiopsis* (Burkhardt et al., 2023), and the *Pleurobrachia* tentacle pocket (Norekian and Moroz, 2019b). These ultrastructural data provide additional support for the convergent nature of ctenophore neurons (Moroz et al., 2014; Moroz and Kohn, 2016).
2. Unique tripartite synapses, unique molecular neural and synaptic toolkits, unique expression of transcription factors, and diversity of unique ctenophore-specific neuropeptides, plus deficiency of bilaterian + cnidarian low molecular weight transmitters, are arguments for the hypothesis of independent origins of ctenophore neural systems, as proposed earlier (Moroz, 2009; Moroz, 2014; Moroz et al., 2014; Moroz, 2021).
3. Whether the syncytial organization of some ctenophore larval neurons is a primarily or secondary traits remains to be determined by ongoing comparative analyses of other ctenophore species. More likely, neuroid syncytia are evolutionarily derived events and relatively rare specializations for particular functions, as evident from other fused neurons in some cnidarians and bilaterians.
4. The directionality of neuronal signaling in ctenophores is evident from the behaviors of these animals as ambush or active predators. Existing information favors the predominance of chemical signaling in ctenophores and its essential role in neuronal integration and behavioral control to be further explored. For example, suppression of synaptic transmission in high magnesium solutions eliminated the coordinated activity of cilia in intact and semi-intact ctenophore preparations (Norekian and Moroz, 2023).
5. The emerging peptidergic nature of the ctenophore neural systems (Moroz et al., 2014; Sachkova et al., 2021; Hayakawa et al., 2022) is consistent with the hypothesis that neurons evolved from secretory cells (Moroz, 2009; 2014; 2021). Moreover, the astonishing diversity and higher information capacity of classical synapses (Moroz and Romanova, 2021) and volume transmission indicate that chemical signaling is the hallmark of neural and other integrative systems regardless of their origins (Moroz et al., 2021b; Norekian and Moroz, 2021; Moroz and Romanova, 2022). Cajal’s neuronal doctrine applies to ctenophores in full and “…could sustain *some exceptions*” (Cajal, 1995; Bullock et al., 2005) as secondary specializations to be investigated functionally.
6. Finally, in addition to neuronal systems, the ctenophore evolved several parallel electrical conductive systems in the ciliated furrows via gap junctions formed by at least 12 innexins (Hernandez-Nicaise, 1968; Tamm, 1973; Tamm, 1982; Tamm, 1984; Tamm and Moss, 1985; Hernandez-Nicaise, 1991; Tamm and Tamm, 2002; Moroz et al., 2014; Tamm, 2014; Moroz and Kohn, 2016; Norekian and Moroz, 2020). We might also expect the presence of alternative integrative (electrical and chemical) systems in this still enigmatic group of early-branching metazoans.
